# The effect of proliferative hypertrophic scars on determining treatment options for preventing recurrence of vesicourethral anastomotic stenosis after radical prostatectomy: a single-center cross-sectional study

**DOI:** 10.1590/1516-3180.2020.0349.R1.28012021

**Published:** 2021-04-26

**Authors:** Ismail Selvi, Ali Ihsan Arik, Mehmet Sinan Basay, Halil Basar

**Affiliations:** I MD. Physician, Department of Urology, Department of Urology, Karabük University Training and Research Hospital, Karabük, Turkey; II MD. Physician, Department of Urology, Health Science University Dr. Abdurrahman Yurtaslan, Ankara Oncology Training and Research Hospital, Ankara, Turkey.; III MD. Physician, Department of Urology, Health Science University Dr. Abdurrahman Yurtaslan, Ankara Oncology Training and Research Hospital, Ankara, Turkey.; IV MD. Professor, Department of Urology, Health Science University Dr. Abdurrahman Yurtaslan, Ankara Oncology Training and Research Hospital, Ankara, Turkey.

**Keywords:** Urinary bladder neck obstruction, Prostatectomy, Cicatrix, hypertrophic, Proliferative hypertrophic scar, Retropubic radical prostatectomy, Vancouver scar scale, Vesicourethral anastomotic stenosis

## Abstract

**BACKGROUND:**

Vesicourethral anastomotic stenosis (VUAS) following retropubic radical prostatectomy (RRP) significantly worsens quality of life.

**OBJECTIVES:**

To investigate the relationship between proliferative hypertrophic scar formation and VUAS, and predict more appropriate surgical intervention for preventing recurrent VUAS.

**DESIGN AND SETTING:**

Retrospective cross-sectional single-center study on data covering January 2009 to December 2019.

**METHODS:**

Among 573 male patients who underwent RRP due to prostate cancer, 80 with VUAS were included. They were divided into two groups according to VUAS treatment method: dilatation using Amplatz renal dilators (39 patients); or endoscopic bladder neck incision/resection (41 patients). The Vancouver scar scale (VSS) was used to evaluate the characteristics of scars that occurred for any reason before development of VUAS.

**RESULTS:**

Over a median follow-up of 72 months (range 12-105) after RRP, 17 patients (21.3%) had recurrence of VUAS. Although the treatment success rates were similar (79.5% versus 78.0%; P = 0.875), receiver operating characteristic (ROC) curve analysis indicated that dilatation using Amplatz dilators rather than endoscopic bladder neck incision/resection in patients with VSS scores 4, 5 and 6 may significantly reduce VUAS recurrence. A strong positive relationship was observed between VSS and total number of VUAS occurrences (r: 0.689; P < 0.001). VSS score (odds ratio, OR: 5.380; P < 0.001) and time until occurrence of VUAS (OR: 1.628; P = 0.008) were the most significant predictors for VUAS recurrence.

**CONCLUSIONS:**

VSS score can be used as a prediction tool for choosing more appropriate surgical intervention, for preventing recurrent VUAS.

## INTRODUCTION

The common feature of urethral stricture, bladder neck stenosis and surgical incision scars is that they develop due to poor wound healing.^1^^,^^2^ In the pathogenesis of these disorders, many mediators and molecules such as transforming growth factor-β1, basic fibroblast growth factor and platelet-derived growth factor play a role. These conditions develop as a result of chronic inflammation.^3^

The rate of hypertrophic scar development in the whole population has been reported to be 1.5-4.5%.^4^ Although the anterior chest wall and posterior ear are the most common sites for hypertrophic scar formation among anatomical regions, these scars may also be commonly seen elsewhere in the upper body. Individual predisposition, various genetic and hereditary factors and various systemic diseases may also facilitate development of proliferative hypertrophic scars.^4^

Although some studies have shown that the presence of hypertrophic scars may be an independent factor for predicting the development of urethral stenosis, we could not find any study that had directly investigated the relationship between proliferative hypertrophic scar formation and vesicourethral anastomotic stenosis (VUAS) after retropubic radical prostatectomy (RRP).

## OBJECTIVES

Our aim was to evaluate the existence of this relationship. In addition, we aimed to predict which surgical intervention for VUAS might be more appropriate for preventing recurrent stenosis, depending on the degree of proliferative hypertrophic scar formation.

## METHODS

### Patients and study design

Our study was designed as a cross-sectional study after obtaining approval from the local ethics committee (protocol number: 77192459-050.99-E.12077 - 7/35; date of approval: November 12, 2019) and written informed patient consent. A total of 573 male patients aged 56-74 years who underwent open RRP due to prostate cancer, operated by the same surgical team between January 2009 and December 2019, were retrospectively evaluated. Our study was conducted in accordance with the ethical standards of the institutional and/or national research committee and with the 1964 Helsinki declaration and its later amendments or comparable ethical standards. We also used a checklist in accordance with the STROBE recommendations (STrengthening the Reporting of OBservational studies in Epidemiology).

Patient demographic data, comorbidities, medical treatments, previous surgeries and clinical data relating to prostate cancer were recorded. Intraoperative and postoperative complications following radical prostatectomy, the history of VUAS, the time when VUAS developed and the surgical method that was used to treat VUAS were recorded. Data relating to scar status were obtained during routine three-month postoperative follow-up examinations. Among the patients with complete data, those who had previously developed incision scar formation for any reason before surgical intervention to treat VUAS were included in the study. The exclusion criteria are listed below:

- Patients with missing data relating to incision scar formation and postprostatectomy follow-up period
- Patients who underwent any bladder, prostate or urethral operation prior to radical prostatectomy or development of VUAS
- Patients who received radiotherapy in the pelvic region for any reason prior to radical prostatectomy
- Patients without incision scar formation on the body for any reason before VUAS
- Patients with a history of urethral stricture or bladder neck stenosis prior to radical prostatectomy or those with urethral stricture concomitant with VUAS

**Figure 1.**
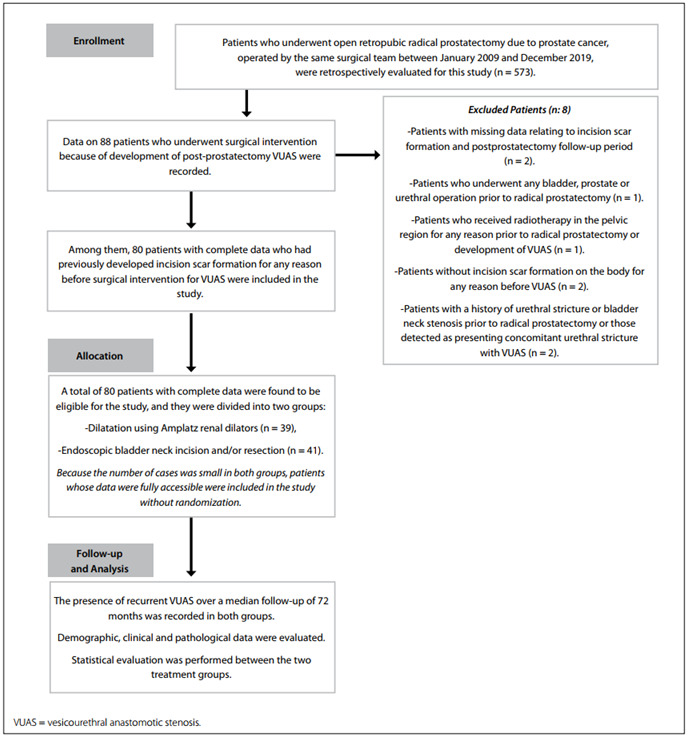
Flowchart of the study population.

A flowchart of the study population is shown in Figure 1.

### Vancouver scar scale

This scale evaluates the characteristics of scars in terms of vascularity, height/thickness, pliability and pigmentation. It was first described by Sullivan in 1990, for burn scar assessment.^5^ The patient’s subjective perception of the scars is not included in the general score. The scoring on this scale is as follows:

Vascularity: normal (0), pink (1), red (2), purple (3)
Pigmentation: normal (0), hypopigmentation (1), hyperpigmentation (2)
Pliability: normal (0), supple (1), yielding (2), firm (3), ropes (4), contracture (5)
Height: flat (0), ≤ 2 mm (1), 2-5 mm (2), ≥ 5 mm (3)
The total score can range from 0 to 13.

The patients were divided into two groups according to the treatment method for VUAS. The first group consisted of 39 patients who underwent dilatation using Amplatz renal dilators. The second group consisted of 41 patients who underwent endoscopic bladder neck incision and/or resection. Any presence of recurrent stenosis during the follow-up was recorded in both groups.

### Surgical procedure for vesicourethral anastomosis in retropubic radical prostatectomy

A non-bladder neck sparing approach was used to remove the prostate. A 2/0 absorbable multifilament suture in a ‘tennis racquet’ fashion, to a size of 22 French (Fr), was used for bladder neck reconstruction. After the mucosa had been everted over the bladder neck with 4/0 absorbable sutures, vesicourethral anastomosis was performed using six 1/0 absorbable multifilament sutures at the 2, 4, 6, 8, 10 and 12 o’clock positions, over a 20 Fr Foley catheter. The urethral catheter was left in place for three weeks.

### Dilatation technique using Amplatz renal dilators

Under regional or local anesthesia and in the lithotomy position, a 0.038-inch stiff hydrophilic guidewire was manipulated beyond the stenosis, through cystoscopy, and was advanced into the bladder. Sequential dilatation was performed using Amplatz renal dilators from 10F to 26F with an 8F stylet. During the dilatation, the Amplatz dilators were advanced by means of rotation towards the bladder with use of a lubricant. After dilatation, an 18 Fr urethral catheter was inserted with guidance through a guidewire and was maintained there for 5 days. A three-month self-catheterization protocol was recommended after removal of the catheter.

### Technique for endoscopic bladder neck incision and/or resection

Under regional anesthesia and in the lithotomy position, the narrow-sclerosis part of the bladder neck was incised with diathermic incisions at the 4, 8 and 12 o’clock positions with 2-4 radial incisions. The incisions were made as far as the perivesical area of the bladder neck. If necessary, sclerotic areas of the bladder neck were resected with a 26 F resectoscope. These resections were performed deeply at the 3 and 9 o’clock positions. After both procedures, an 18 Fr urethral catheter was inserted and maintained there for 5 days. A three-month self-catheterization protocol was recommended after removal of the catheter.

### Diagnosis of postoperative vesicourethral anastomotic stenosis

The patients presented with complaints such as weak urinary flow rate, dripping after urination, incontinence, residual urinary sensation and inability to completely drain the bladder after radical prostatectomy. Occurrences of weak urinary flow (Qmax < 10 ml/sec) were determined through uroflowmetry and post-micturition residual urine through ultrasonography. Retrograde urethrography was used to make the differential diagnosis between VUAS and concomitant urethral stricture and to identify the location and length of stenosis.

Because contrast did not adequately pass the proximal urethra or bladder neck in cases of very tight stenosis, voiding cystourethrography was performed if necessary, by passing a small feeding tube into the bladder. Alternatively, anterograde urethrography was performed by placing a suprapubic tube. Urodynamic testing was performed to evaluate bladder capacity, compliance and detrusor contractility whenever there was suspicion of bladder dysfunction.

**Figure 2.**
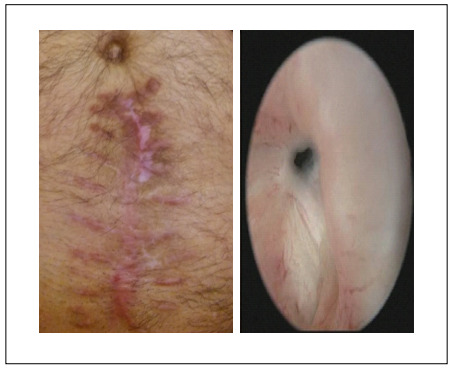
Incision scar (Vancouver scar scale = 8) and cystoscopy images from a single patient.

The definitive diagnosis was made by means of cystourethroscopy, using a 17 Fr cystoscope. Although urethral dilatation to 22 Fr via a catheter was tried as the initial management, endoscopic intervention was required in resistant cases. Incision scar and cystoscopy images from a single patient are shown in Figure 2.

Surgical success in both groups was defined as having no evidence of recurrence (Qmax more than 15 ml/sec; post-micturition residual urine < 50 ml) at the 1^st^, 3^rd^, 6^th^ and 12^th^ postoperative months and every three-month follow-up. Obstructive symptoms, Qmax smaller than 10 ml/sec and any need for repeated surgical urethral interventions were defined as recurrence.

### Statistical analyses

The Kolmogorov-Smirnov and Shapiro-Wilk tests were used to evaluate normality of distribution. An independent-sample t test was used to detect differences between two groups of normally distributed variables, and the Mann-Whitney U test was performed for non-normal distribution. The chi-square test was used for categorical variables. The relationship between variables was assessed by means of the Spearman correlation test. Receiver operating characteristic (ROC) curve analysis was performed to determine cutoff values for Vancouver scar scale (VSS) scores in order to predict recurrent VUAS. Binary logistic regression analysis was used to determine the predictive factors for recurrence of VUAS. P-values of < 0.05 were considered statistically significant. All the statistical analyses were performed using IBM SPSS Statistics v23 (IBM, Armonk, NY, United States).

## RESULTS

Among all the 573 patients who underwent RRP, VUAS developed in 88 patients (15.3%). For 80 patients with a median age of 66 years (range 56-74), complete data were available, and these patients were included in our study. Demographic, clinical and pathological data on these patients are shown in Table 1. During the median follow-up of 72 months (range 12-105) after RRP, 17 patients (21.3%) had recurrence of VUAS. None of these patients complained of severe urinary incontinence. Pelvic floor exercises were enough to relieve the symptoms in most patients.

**Table 1.** Demographic, clinical and pathological data and oncological outcomes of the patients

Parameters	Group I (n = 39)	Group II (n = 41)	Total (n = 80)	P-value
Median age (25^th^ 75^th^ percentile) minimum-maximum	68.00 (64.00-69.00) 56-74	66.00 (63.00-69.50) 57-74	66.00 (63.00-69.00) 56-74	0.367^§^
Median BMI (25^th^ 75^th^ percentile)	25.20 (23.10-27.80)	24.50 (21.95-27.10)	24.65 (22.35-27.57)	0.174^§^
Median preoperative PSA level (25^th^ 75^th^ percentile)	7.44 (5.19-9.60)	8.95 (6.38-11.00)	8.00 (6.03-10.34)	0.115^§^
Biopsy Gleason grade (n, %)				
-6 -7 -8 -9 -10	30 (76.9) 5 (12.9) 2 (5.1) 2 (5.1) 0 (0.0)	26 (63.5) 6 (14.6) 6 (14.6) 2 (4.9) 1 (2.4)	56 (70.0) 11 (13.7) 8 (10.0) 4 (5.0) 1 (1.3)	0.504^‡^
Preoperative clinical T stage (n, %)				
-T1c -T2a -T2b -T2c -T3a	24 (61.5) 7 (17.9) 1 (2.6) 6 (15.4) 1 (2.6)	31 (75.6) 4 (9.8) 3 (7.3) 2 (4.9) 1 (2.4)	55 (68.7) 11 (13.8) 4 (5.0) 8 (10.0) 2 (2.5)	0.324^‡^
Post-prostatectomy Gleason grade (n, %)				
-6 -7 -8 -9 -10	20 (51.3) 10 (25.6) 5 (12.8) 4 (10.3) 0 (0.0)	23 (56.1) 7 (17.1) 8 (19.5) 0 (0.0) 3 (7.3)	43 (53.8) 17 (21.3) 13 (16.3) 4 (5.0) 3 (3.8)	0.894^‡^
Post-prostatectomy pathological T stage (n, %)				
-T2a -T2b -T2c -T3a -T3b	8 (20.5) 5 (12.8) 13 (33.4) 6 (15.4) 7 (17.9)	18 (43.9) 4 (9.8) 8 (19.5) 5 (12.2) 6 (14.6)	26 (32.5) 9 (11.1) 21 (26.3) 11 (13.8) 13 (16.3)	0.261^‡^
Surgical margin positivity (n, %)				
-Yes -No	4 (10.3) 35 (89.7)	7 (17.1) 34 (82.9)	11 (13.8) 69 (86.3)	0.376^‡^
Post-prostatectomy PSA recurrence (n, %)				
-Yes -No	12 (30.8) 27 (69.2)	14 (34.1) 27 (65.9)	26 (32.5) 54 (67.5)	0.747^‡^
Median pre-prostatectomy ASA score (25^th^ 75^th^ percentile)	2.00 (2.00-3.00)	2.00 (1.50-3.00)	2.00 (2.00-3.00)	0.745^§^
Median pre-prostatectomy ACCI score (25^th^ 75^th^ percentile)	5.00 (4.00-6.00)	5.00 (5.00-6.00)	5.00 (4.00-6.00)	0.437^§^
Presence of preoperative hypertension (n, %)				
-Present -Absent	21 (53.8) 18 (46.2)	18 (43.9) 23 (56.1)	39 (48.8) 41 (51.2)	0.374^‡^
Presence of preoperative diabetes mellitus (n, %)				
-Present -Absent	17 (43.6) 22 (56.4)	16 (39.0) 25 (61.0)	33 (41.3) 47 (58.8)	0.678^‡^
Presence of smoking (n, %)				
-Present -Absent	20 (51.3) 19 (48.7)	23 (56.1) 18 (43.9)	43 (53.8) 37 (46.3)	0.666^‡^
Intraoperative excessive blood loss (> 1000 ml) (n, %)				
-Present -Absent	13 (33.3) 26 (66.7)	15 (36.6) 26 (63.4)	28 (35.0) 52 (65.0)	0.761^‡^
Prolonged leakage at the anastomotic site (> 100 ml in the drainage tube) (n, %)				
-Present -Absent	9 (23.1) 30 (76.9)	12 (29.3) 29 (70.7)	21 (26.2) 59 (73.8)	0.529^‡^
Q max prior to operation for VUAS (ml/s)	8.03 ± 1.53	7.74 ± 1.09	7.88 ± 1.33	0.338^†^
Postvoid residual volume prior to operation for VUAS (ml)	92.97 ± 9.67	90.02 ± 10.04	91.46 ± 9.91	0.185^†^
Median time until occurrence of VUAS (months) (25^th^ 75^th^ percentile)	9.00 (6.00-12.00)	10.00 (7.00-12.00)	9.00 (7.00-12.00)	0.433^§^
Recurrence of VUAS (n, %)				
-Present -Absent	8 (20.5) 31 (79.5)	9 (22.0) 32 (78.0)	17 (21.3) 63 (78.7)	0.875^‡^
Median time until the recurrence of VUAS (months) (25^th^ 75^th^ percentile)	7.00 (6.00-8.75)	9.00 (5.00-10.00)	8.00 (6.00-10.00)	0.373^§^
Median total number of occurrences of VUAS (25^th^ 75^th^ percentile) minimum-maximum	1.00 (1.00-2.00) 1-3	1.00 (1.00-2.00) 1-3	1.00 (1.00-2.00) 1-3	0.489^§^
Median VSS score (25^th^ 75^th^ percentile)	5 (4-6)	4 (3-6)	4 (4-6)	0.083^§^
Median total follow-up period (months) (25^th^ 75^th^ percentile) minimum-maximum	79.00 (23.00-84.00) 12-105	67.00 (62.00-78.00) 18-99	72.00 (57.75-82.75) 12-105	0.467^§^

ACCI = age-adjusted Charlson comorbidity index; ASA = American Society of Anesthesiologists; BMI = body mass index; PSA = prostate-specific antigen; VSS = Vancouver scar scale, VUAS = vesicourethral anastomotic stenosis.

Group I comprises patients who underwent dilatation using Amplatz renal dilators; Group II comprises patients who underwent endoscopic bladder neck incision and/or resection”

^§^Mann-Whitney U test; data are expressed as “median (25^th^ percentile-75^th^ percentile)”; ^‡^Chi-square test; data are expressed as “number (percent)”; ^†^independent-sample t test; data are expressed as “mean ± standard deviation”;

P < 0.05 indicates statistical significance; however, there are no significant values in this table.

There was no significant difference between the two treatment groups in terms of demographic data, clinical data or oncological outcomes (Table 1). Dilatation using Amplatz renal dilators and endoscopic bladder neck incision and/or resection were found to have similar success rates for preventing recurrence of VUAS (79.5% versus 78.0%; P = 0.875, respectively). There was also no significant difference between the groups in terms of the time until the occurrence of VUAS (9 versus 10 months; P = 0.433, respectively) and the time until the recurrence of VUAS (7 versus 9 months; P = 0.373, respectively).

The cutoff values of VSS scores for predicting recurrence of VUAS in the two groups are shown in Table 2. According to these results, patients with a VSS score > 3.5 were more likely to have recurrence of VUAS if endoscopic bladder neck incision and/or resection was performed. Conversely, patients with a VSS score > 6.5 were more likely to have recurrence of VUAS if dilatation using Amplatz renal dilators was performed. Although there was no significant difference between the groups in terms of median VSS scores, we observed a strong positive relationship between VSS and total number of occurrences of VUAS (rho: 0.689; P < 0.001). A moderate negative correlation was also observed between VSS and the time until the occurrence of VUAS (rho: -0.530, P < 0.001), but no relationship was found between VSS and time until recurrence of VUAS (rho: -0.310; P = 0.115).

**Table 2.** Cutoff values of Vancouver scar scale for predicting stenosis recurrence following two different types of operation for treating for vesicourethral anastomotic stenosis

	Dilatation using Amplatz renal dilators	Endoscopic bladder neck incision and/or resection
Cutoff value	6.5	3.5
Sensitivity (%)	87.3	93.3
Specificity (%)	84.6	80.8
Positive predictive value (%)	85.0	82.9
Negative predictive value (%)	86.9	92.3
Area under receiver operating characteristic curve	0.920	0.949
P	< 0.001^*^	< 0.001^*^

^*^P < 0.05; Asterisk (*) indicates statistical significance.

In multivariate analysis, VSS score (OR: 5.380; P < 0.001) and time until occurrence of VUAS (OR: 1.628; P = 0.008) were found to be the most significant determinants for predicting recurrence of VUAS (Table 3).

**Table 3.** Factors predicting recurrence of post-prostatectomy vesicourethral anastomotic stenosis

	Univariate model				Multivariate model			
	OR	95% CI		P	OR	95% CI		P
		Lower	Upper			Lower	Upper	
Patient’s age	1.095	0.986	1.218	**0.090**				
BMI	1.061	0.932	1.208	**0.373**				
Preoperative PSA level	1.027	0.905	1.164	**0.683**				
Biopsy Gleason grade	1.418	0.881	2.283	**0.150**				
Preoperative clinical T stage	1.517	0.909	2.531	**0.110**				
Post-prostatectomy Gleason grade	1.020	0.671	1.552	**0.924**				
Post-prostatectomy pathological T stage	1.128	0.819	1.555	**0.459**				
Surgical margin positivity	1.515	0.368	6.250	**0.565**				
Post-prostatectomy PSA recurrence	1.727	0.619	4.807	**0.296**				
Pre-prostatectomy ASA score	1.044	0.544	2.004	**0.894**				
Pre-prostatectomy ACCI score	1.225	0.923	1.626	**0.160**				
Presence of preoperative hypertension	1.440	0.570	3.636	**0.440**				
Presence of preoperative diabetes mellitus	1.387	0.547	3.512	**0.491**				
Presence of smoking	1.763	0.841	5.448	**0.266**				
Qmax prior to operation for VUAS (ml/s)	1.164	0.820	1.652	**0.395**				
Postvoid residual volume prior to operation for VUAS (ml)	1.004	0.958	1.052	**0.850**				
Operation type for VUAS	1.154	0.460	2.896	**0.761**				
Time until the occurrence of VUAS (months)	1.865	1.420	2.457	< 0.001^*^	1.628	1.137	2.331	0.008^*^
VSS score	5.380	2.641	10.958	< 0.001^*^	5.380	2.641	10.958	< 0.001^*^

OR = hazard ratio; CI = confidence interval; BMI = body mass index; PSA = prostate-specific antigen; ASA = American Society of Anesthesiologists; ACCI = age-adjusted Charlson comorbidity index; VUAS = vesicourethral anastomotic stenosis; VSS = Vancouver scar scale; Qmax: peak flow rate.

^*^P < 0.05; Asterisk (*) indicates statistical significance.

## DISCUSSION

The incidence of VUAS after open RRP has been found to range from 0.4% to 32% in different series.^6^^,^^7^ The incidence has been decreasing through the help of surgical techniques and new technological developments over recent years.^8^ The rate of VUAS was found to be 1.1% after robotic assisted laparoscopic radical prostatectomy (RALP), whereas it was reported as 4.7% in a series that underwent laparoscopic radical prostatectomy.^9^ Most studies have only reported on the patients who underwent treatment for VUAS, so the rates in the literature are considered to be lower than the true incidence.^10^ Although there are studies reporting that the development of VUAS is significantly reduced by means of bladder neck protection methods during RALP,^11^ there are also contradictory findings suggesting that preservation of the bladder neck reduces VUAS rates.^10^^,^^12^ Symptomatic VUAS is usually seen within six months following RRP.^13^^,^^14^ In our series, it was observed with a median follow-up of nine months.

Vesicourethral anastomotic stenosis may develop due to fibrosis in the anastomosis line between the bladder neck and urethra. It is thought that tension in anastomosis, postoperative hemorrhage, large-volume blood loss, pelvic hematoma formation, urinary leakage from the anastomosis, disrupted peri-bladder neck vascular supply, overnarrowing of the bladder neck during anastomosis, prolonged catheterization, acute retention after urethral catheter removal, prior radiation or a history of hypertrophic scar formation may cause VUAS.^6^^,^^8^^,^^13^ These factors may cause a peri-anastomotic inflammatory response that results in scar formation.^6^ In addition, in the presence of obesity, diabetes mellitus, smoking, advanced age or vascular disease, susceptibility to VUAS increases since there is no adequate microvascular environment for anastomotic healing.^6^^,^^15^ The surgeon’s experience is also one of the known factors playing a role in complications after RRP, and this may affect VUAS rates.^6^ In different open RRP series, the VUAS rates have been reported to be 19.8%-22% for high-volume surgeons.^14^^,^^16^ In our study, all the operations were performed by the same surgical team (approximately 45 RRP procedures per year) and, thus, we tried to exclude the effect of the surgeon’s experience.

The most commonly used first-line surgical methods for treating VUAS are dilatation with urethral catheters, rigid bougie dilators or urethral balloon dilators, dilatation using Amplatz renal dilators and endoscopic bladder neck incision or resection.^17^ In cases of failure, with repeated interventions such as internal urethrotomy, metallic urethral stent or endourethroplasty using interstitial injection of antiproliferative agents, open surgical treatments may be recommended as the last option for treatment.^13^^,^^18^ Adding this undesirable complication to the anxiety and mood disorder caused by prostate cancer may affect the person’s life even more negatively.

Guidewire-assisted dilatation avoids the complication rates associated with blind dilatation techniques such as false passage, incontinence, impotence or rupture of the rectum.^13^^,^^19^ Therefore, Amplatz renal dilators that are used for tract dilatation in percutaneous renal surgeries have begun to be used for dilatation of urethra or bladder neck strictures, as an alternative method.^19^ The success rates for this technique have been reported as 73-92.3% at 21-month follow-up.^19^ According to our results, the recurrence rates were 20.5% and 21.9%, respectively, in patients who underwent dilatation using Amplatz renal dilators and endoscopic bladder neck incision and/or resection at a median follow-up of 72 months.

The presence of a poorly healed median sternotomy incision scar has also been shown to be associated with poor wound healing in urethral tissue. It has been reported that patients with advanced median sternotomy scars develop longer segmented and frequently recurrent urethral stenosis after urethral manipulations.^20^ There have not been enough studies investigating any similar relationship with VUAS, but we observed a strong positive relationship between VSS and development and recurrence of VUAS. Although a cutoff value for the VSS score has not been defined for a description of hypertrophic scars, the most accepted score has been 4, in various studies.^20^^,^^21^^,^^22^ A maximal abdominal scar width > 10 mm has been found to have an eight-fold greater likelihood of VUAS after open RRP.^8^ In accordance with this information, we observed that median VSS scores were higher in patients with recurrence of VUAS (6 versus 3; P < 0.001).

Some authors have stated that although urethral dilatation and endoscopic laser incision and/or resection can significantly cure VUAS, residual fibrotic tissue may be left. Using electrocautery to provide hemostasis has also been associated with new fibrosis triggered by thermal damage.^13^^,^^19^ This may be a risk factor for development of new fibrosis and recurrent stenosis.^13^ Although holmium laser incision or plasma-button vaporization of VUAS has been reported to have significantly higher success rates,^13^^,^^14^ there have also been studies contradicting this, in which it was reported that there were no significant differences in the results from the holmium laser, electrocautery or cold knife incision.^23^ According to our findings, dilatation using Amplatz renal dilators gave rise to less risk of recurrent fibrotic stenosis than did diathermic incision and resection, even in patients with high susceptibility to development of proliferative hypertrophic scars.

The rate of recalcitrant VUAS with more than three unsuccessful endoscopic interventions has been reported to be 25-30%.^24^^,^^25^ The treatment options should be carefully chosen in cases of refractory stenosis. Patient factors relating to comorbidities, previous interventions or complications, and surgical factors relating to morbidity, surgical expertise or requirement of reconstructive surgical experience need to be taken into account. Therefore, it is important to choose a method in which the likelihood of recurrence may be lower in these patients. Pfalzgraf et al.^26^ stated that VUAS recurrence after transurethral incision or transurethral resection was not predictable. In our study, we aimed to predict the more appropriate surgical intervention for preventing recurrent VUAS. Although the two treatments had similar success rates, it seemed that dilatation using Amplatz renal dilators, rather than endoscopic bladder neck incision and/or resection, in patients with VSS scores of 4, 5 and 6 could significantly reduce VUAS recurrence. Contrary to the opinion of Pfalzgraf et al.,^26^ our findings showed that it can be predicted which surgical intervention is more appropriate for preventing recurrent VUAS.

When minimally invasive interventions such as endoscopic interventions or dilatation techniques fail, repetition of these interventions subsequently may substantially reduce the success rate of future reconstructive surgeries. Therefore, prediction of VUAS cases with a high likelihood of recurrence may lead to preference of open or perineoscopic reconstruction operations initially.^27^ Through this, success rates may further increase and patients may avoid repeated procedures. Although our findings did not allow us to make such estimates directly, our preliminary results suggest that use of VSS in a particular patient group may help predict the surgical intervention method that is more appropriate for preventing VUAS recurrence.

To the best of our knowledge, this was the first study to investigate the relationship between proliferative hypertrophic scar formation and the success of treatment options for VUAS following open RRP. Most studies in the literature have limitations because of small patient populations, retrospective design, heterogeneous patient groups with concomitant urethral strictures and variable definitions of VUAS. Although most studies mentioned above have described absence of recurrence in the first six months after intervention as a criterion for success,^13^^,^^14^^,^^25^^,^^28^ we found that any recurrence requiring additional surgical procedures, regardless of the time that had elapsed, was indicative of failure.

Radiotherapy that is used for adjuvant or salvage treatment is also a predisposing factor for development of necrosis and fibrosis of the bladder neck, due to progressive obliterative endarteritis.^29^ In our study, we excluded patients who had undergone radiotherapy, and also those with urethral stricture or bladder neck stenosis prior to RRP, in order to form a homogeneous patient group.

Taking a critical view, the utility of VSS may not seem significant enough, given that minimally invasive surgical techniques for prostate cancer that reduce the rate of VUAS have been developed. Nonetheless, although minimally invasive techniques do not give rise to incision lines that are as long as in open surgery, proliferative hypertrophic scars may also develop at the trocar sites in these methods. Therefore, although the rate of VUAS is decreased through minimally invasive surgery, it is still likely to be seen. Based on the relationship between proliferative hypertrophic scar formation and VUAS in our study, we think that our findings may also guide clinicians in choosing a more appropriate surgical intervention that can reduce the likelihood of recurrence, even in cases of VUAS following minimally invasive surgery.

Although our study revealed a novel prediction, its retrospective nonrandomized design with a limited number of patients and a relatively short follow-up period in a single center is its main limitation. Moreover, although our VUAS rates were consistent with traditional open RRP rates, they were higher than what is seen in robotic surgery data, and are too high for a modern series. In addition, performing open RRP despite the current technological advances can be considered to be a serious limitation in this era of uro-technology. On the other hand, robotic surgery is not available in every institution, because of various financial reasons. Therefore, open surgery still remains a commonly used technique in many developing countries, even though it has been reported to have a tenfold greater risk of development of VUAS, compared with minimally invasive surgery. Furthermore, we only compared the two most-used treatment methods in our department. Other popular methods, such as endoscopic holmium laser incision, plasma button vaporization and injection of antifibrotic agents or steroids into the incision site were not used in this study. We also were unable to evaluate the relationship between the success rates of these methods and the presence of proliferative hypertrophic scars. Lastly, in similar studies in the literature, patients with a scar on the anterior chest wall were included because this body area is more likely to develop hypertrophic scar formation. However, we also included patients who had previously developed incision scars for any reason, in areas other than this one. Although we evaluated these scars through VSS, this can be considered to be a limitation in terms of standardization.

## CONCLUSION

The VSS score can be used as a prediction tool for choosing a more appropriate method for individualized treatment among patients at higher risk of scarring. We present our findings as “preliminary results” because it was not easy to obtain comprehensive results, due to the limitations of our study. Nevertheless, because the relationship between proliferative hypertrophic scar formation and development of VUAS had not been investigated before, we think that our preliminary results may be a step towards further studies.
